# Ultraviolet Light Exposure Decreases Thyroid Cancer Risk: A National Perspective

**DOI:** 10.3390/biomedicines10102452

**Published:** 2022-10-01

**Authors:** Tessa R. Lavorgna, Mohammad Hussein, Peter P. Issa, Eman Toraih, Emad Kandil

**Affiliations:** 1 School of Medicine, Tulane University, New Orleans, LA 70112, USA; 2 Department of Surgery, School of Medicine, Tulane University, New Orleans, LA 70112, USA; 3 School of Medicine, Louisiana State University Health Sciences Center, New Orleans, LA 70112, USA; 4 Genetics Unit, Department of Histology and Cell Biology, Faculty of Medicine, Suez Canal University, Ismailia 41522, Egypt

**Keywords:** ultraviolet light, SEER program, disparity, thyroid cancer

## Abstract

Ultraviolet (UV) light has been reported to have both pro-oncogenic and anti-oncogenic effects. Since patient pigmentation can influence the role of UV light exposure, we thought to investigate the recent trends in thyroid cancer incidence and survival with an emphasis on patient race and UV exposure. Patients diagnosed with thyroid cancer from the Surveillance, Epidemiology, and End Results (SEER) database were identified. A total of 284,178 patients were enrolled. Data were stratified according to population sex, race, and state. UV exposure data in Watt-Hours Per Square Meter for the state were obtained from the National Cancer Institute Cancer Atlas. Thyroid cancer incidence rate varied by race, ranging from 14.9 cases per 100,000 in Asian or Pacific Islanders and 14.7 per 100,000 in Caucasians, to 8.7 per 100,000 in African American and 8.0 per 100,000 in Native Americans. UV exposure was negatively correlated with thyroid cancer incidence when analyzed across all populations (r = −0.299, *p* = 0.035). UV exposure was most steeply negatively correlated with thyroid cancer rates in Black populations (r = −0.56, *p* < 0.001). Despite this, Black men had the worst 5-year survival rate when compared to other ethnic populations. Overall, UV exposure does not increase the risk of thyroid cancer and may serve as a protective factor in the development of thyroid cancer.

## 1. Introduction

Thyroid cancer is the most common endocrine tumor [1] and, with its increased incidence rates over the last 50 years, is the fastest growing cancer in the United States [2]. The number of new cases of thyroid cancer is two-to-three times higher in females than in males [3,4]. Yet, it has been suggested that higher sex-based incidences may be due to the overdiagnosis of clinically silent tumors in females, as sex-based differences in mortality rates do not exist [1].

In addition to the established risk factors of increased body mass index (BMI) and benign thyroid conditions, recent research has examined the role of ultraviolet (UV) exposure in thyroid cancer development [5]. UV light is a non-ionizing form of energy (ionizing light is of higher energy) that is emitted by both solar and artificial (e.g., UV tanning bed) light which can be absorbed by humans when in direct exposure with the skin. Three subtypes of UV light exist: UV-A (wavelengths of 315–399 nm), UV-B (wavelengths of 280–314 nm), and UV-C (wavelengths of 100–279 nm). While UV-B and UV-C are mostly absorbed by the ozone layer and consequently do not reach the Earth’s atmosphere, UV-A levels contribute to a person’s lifetime exposure level. UV exposure presents both beneficial and harmful consequences to humans, and consequently its balanced exposure is critical for human health. On the one hand, UV overexposure significantly increases the risk of developing melanoma owing to induced oxidative stress and mutation of p53 tumor suppressor genes [6]. Conversely, UV light converts vitamin D into its active form, calcitriol, and enhances calcium and phosphorous regulation, bone health, and immune function [7]. In addition, UV exposure, contrary to common dogma, has been shown to be inversely associated with the incidence of solid organ malignancies, including those of the prostate [8,9], female breast [10,11], and ovary [12].

The influence of UV exposure on thyroid cancer incidence and thyroid cancer patient outcomes remains a topic of recent debate. One epidemiological study reported in that ambient daily UV light was positively correlated with an increased risk of both cancerous and benign thyroid disorders [13]. These results were in contrast with a 2017 United-States based prospective study of over 44,000 participants [5]. In the latter study, UV radiation, measured by the time spent outdoors and the patient’s corresponding geographic residential location which corresponded with a satellite-based UV radiation measurement, was associated with a decreased risk of thyroid cancer [5]. However, this study restricted their patient cohort to only White individuals, reducing the generalizability of their work. Furthermore, neither study stratified their outcomes by patient race nor determined patient outcomes (e.g., 5-year survival). Here, we further elucidate the relationship between UV exposure and the risk of thyroid cancer with a particular focus on race-based differences.

## 2. Methods

### 2.1. Study Sample

Overall estimates of thyroid cancer in the United States were retrieved from the National Cancer Institute’s Surveillance, Epidemiology, and End Results (SEER) database from 2001 to 2018. The SEER program publishes data from cancer registries covering roughly 30% of the United States population [14]. Data were stratified according to the population race as well as the state’s geographical location. UV exposure data in Watt-Hours Per Square Meter for the state were obtained from the National Cancer Incidence Cancer Atlas. Since the data were obtained from publicly available databases, institutional review board approval was not required. The SEER database Research Data Agreement was signed before initiating this work. The variables, outcomes, and statistical analyses below were conducted in a manner to work previously published in the field [15,16,17].

### 2.2. Data Variables

Thyroid cancer incidence rates were adjusted by age. Accordingly, age was categorized as <50 years, 50–64 years, and >65 years. To investigate thyroid cancer incidence rates and outcomes by race, we sub-stratified our patient cohort into White, Black, American Indian/Alaskan Native, and Asian or Pacific Islander. Rates for American Indians/Alaska Natives only included cases that were in a Purchased/Referred Care Delivery Area, which is a region in which Indian Health Services deliver care to the American Indians/Alaska Native community living within a designated area (www.seer.cancer.gov/seerstat: accessed date: 15 January 2021). Hispanics and non-Hispanics were not reported to be mutually exclusive from Whites, Blacks, Asian/Pacific Islanders, and American Indians/Alaska Natives. Consequently, incidence data for Hispanics and Non-Hispanics were based on the North American Association of Central Cancer Registries (NAACCR) Hispanic Latino Identification Algorithm, and cases from the Alaska Native Registry were excluded.

Thyroid cancer stage was delineated given the SEER website’s coding summary. Accordingly, “All Stages” referred to any stage of tumor based on the clinical and pathological documentation of the disease, while “late stage” cases were defined as those with regional or distant metastasis.

## 3. Statistical Analysis

A total of 284,178 patients were identified. Age-adjusted incidence rates (cases per 100,000 people per year) were calculated for each state. Data with fewer than 16 records in a specific age–sex–race category stratification were not included in the analysis to ensure the confidentiality and stability of rate estimates, per the SEER guidelines. The relative survival rate was calculated using monthly intervals and the mortality rate was recorded for each cohort. These rates were calculated using SEER*Stat software (version 8.3.6, SEER program, National Cancer Institute, Bethesda, MD, USA). Population counts for denominators of such calculations were based on census populations, as determined by the National Cancer Institute. In addition, the average annual percentage change in thyroid cancer incidence was calculated using the Joinpoint Trend Analysis Software (https://surveillance.cancer.gov/joinpoint/: accessed 1 February 2021) and was based on the annual percentage changes published.

The correlation between the thyroid cancer incidence and mortality rates with average UV exposure in each state was tested using Pearson’s correlation analysis. The correlation coefficient and *p*-value were reported for the overall analysis, and stratification of the analysis was performed by patient sex as well as race/ethnic group. Linear regression analysis was employed to test the role of UV exposure as an independent predictor of thyroid cancer risk across multiple racial/ethnic groups. The percentage of different sexes and race/ethnic populations was adjusted as a confounder. All statistical tests were two sided.

## 4. Results

### 4.1. Thyroid Cancer Incidence by Race/Ethnicity

The overall age-adjusted incidence rate of thyroid cancer was 14.1 cases per 100,000 (Table 1). Thyroid cancers occurred more frequently in women (20.7 cases per 100,000) than compared to men (7.3 per 100,000). When stratified by race, incidence rates varied. Thyroid cancer incidence was most frequent in Asian or Pacific Islanders (14.9 per 100,000), and Whites (14.7 per 100,000), and less frequent in Hispanics (13.4 per 100,000), Blacks (8.7 per 100,000), and Native Americans (8.0 per 100,000). White or Asian/Pacific Islander females had the highest incidence rates in any population, occurring in both groups at 21.7 cases per 100,000. The lowest incidence rates were observed in Black and American Indian/Alaskan Native males, occurring in both groups at 3.8 cases per 100,000.

When analyzing the recent incidence rate trends, the overall incidence rate declined by −2.9 (95% CI: −4.1, −1.7) (Table 1). Recent literature investigating only thyroid malignancies following 2014 have found similar results [18,19]. Moreover, trend analysis found that female incidence rates were decreasing faster than that of males (−3.1 per 100,000 versus −1.9 per 100,000). Differences in trend existed by race, as Black populations had the steepest decline in thyroid cancer incidence rates at −5.4 (95% CI: −7.7, −3.2) and Hispanic patients had the slowest decline (−0.9; 95% CI: −2.7, 1.0). While no overall racial group demonstrated an increasing trend in incidence rates, male American Indian/Alaskan Natives and male Hispanics demonstrated a positive trend. Male American Indian/Alaskan Natives had a trend incidence rate of 3.0 (95% CI: 1.0. 5.1), compared to the 0.7 overall (95% CI: −3.6, 2.4) and −0.3 female (95% CI: −3.9, 3.4) trend incidence rates. Similarly, male Hispanics demonstrated an increasing trend incidence rate of 1.4 (95% CI: 0.3, 2.5), which differed from the −0.9 overall (95% CI: −2.7, 1.0) and −1.0 female (95% CI: −3.0, 1.0) Hispanic trend incidence rates.

### 4.2. Thyroid Cancer 5-Year Survival by Race/Ethnicity

**Figure 1:**
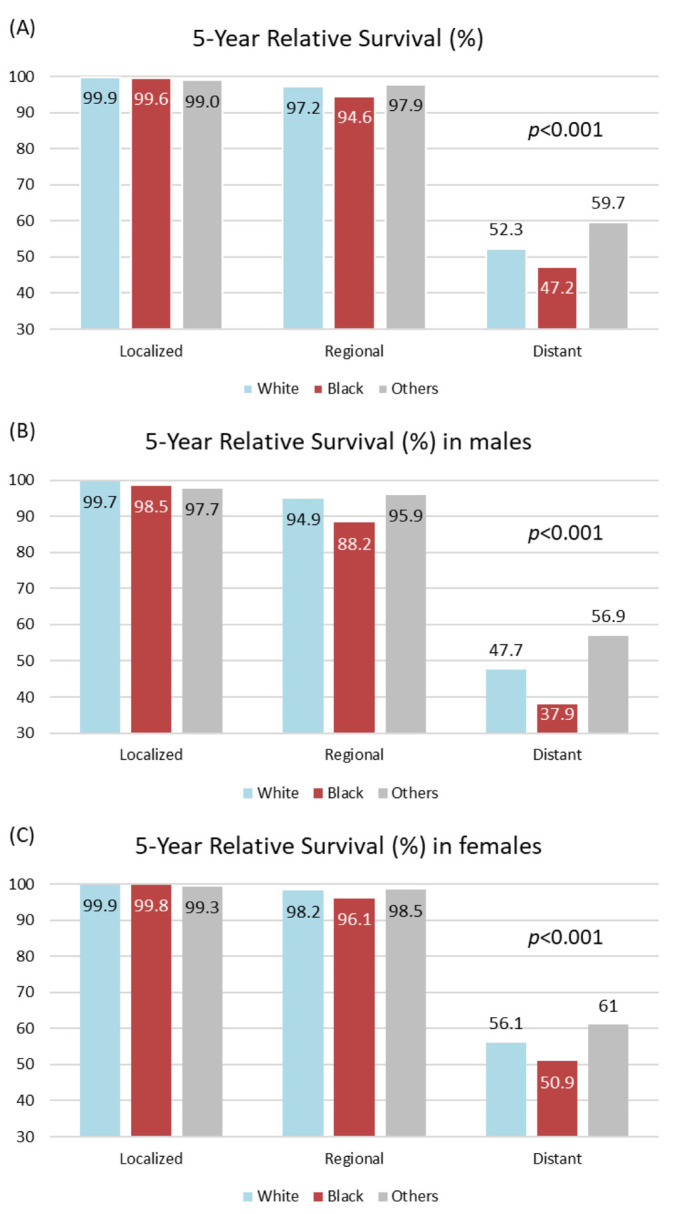
**Survival rate of thyroid cancer patients.** Follow-up of 284,178 patients (71,389 males and 212,789 females) included 237632 White, 22643 Black, and 19204 other races. (**A**) The 5-year relative survival for all cohorts, stratified by cancer stage for both sexes. (**B**) The 5-year relative survival for all cohorts, stratified by cancer stage for males. (**C**) The 5-year relative survival for all cohorts, stratified by cancer stage for females. In SEER, relative survival is defined as the ratio of the proportion of observed survivors in a cohort of cancer patients to the proportion of expected survivors in a comparable set of cancer free individuals. Estimates are based on cases reported by the National Program of Cancer Registries (NPCR) registries from 2011–2017 and follow-up of patients through December 31, 2017. Data are compiled from 42 NPCR registries (Alabama, Alaska, Arizona, Arkansas, California, Colorado, Delaware, Florida, Georgia, Idaho, Illinois, Kansas, Kentucky, Louisiana, Maine, Maryland, Minnesota, Mississippi, Missouri, Montana, Nebraska, Nevada, New Hampshire, New Jersey, New York, North Carolina, North Dakota, Ohio, Oklahoma, Oregon, Pennsylvania, Rhode Island, South Carolina, South Dakota, Tennessee, Texas, Utah, Vermont, Washington, West Virginia, Wisconsin, and Wyoming) that met the data quality criteria for survival analysis, covering approximately 86% of the U.S population. The “Other races” group includes Indian Health Service-linked American Indian, Alaska Native, and Asian and Pacific Islander cases.

Regional and distant thyroid cancer 5-year survival rates differed by sex and race/ethnicity (Figure 1A). In general, men had poorer 5-year relative survival outcomes than females. For example, the survival rate was lower for Black males (88.2%) than for Black females (96.1%) when considering regional thyroid cancers (Figure 1B,C). With respect to race, the regional thyroid cancer 5-year relative survival rates were 97.2% in White populations, 94.6% in Black populations, and 97.9% in all other groups. This discrepancy became significant when analyzing trends in distant thyroid cancers, as the survival rate was 52.3% in Whites, 47.2% in Blacks, and 59.7% in other groups (*p* < 0.001). This finding persisted when sub-grouped by patient sex, with both Black males (37.9%; White: 47.7%; Other: 56.9%; *p* < 0.001) and Black females (50.9%; White: 56.1%; Other: 61.0%; *p* < 0.001) consistently displaying the poorest 5-year survival rates for distant metastases. To investigate this finding further, we analyzed the potential influence of UV exposure.

### 4.3. Ultraviolet Exposure and Thyroid Cancer Incidence Rate by Geographical Correlation

**Figure 2:**
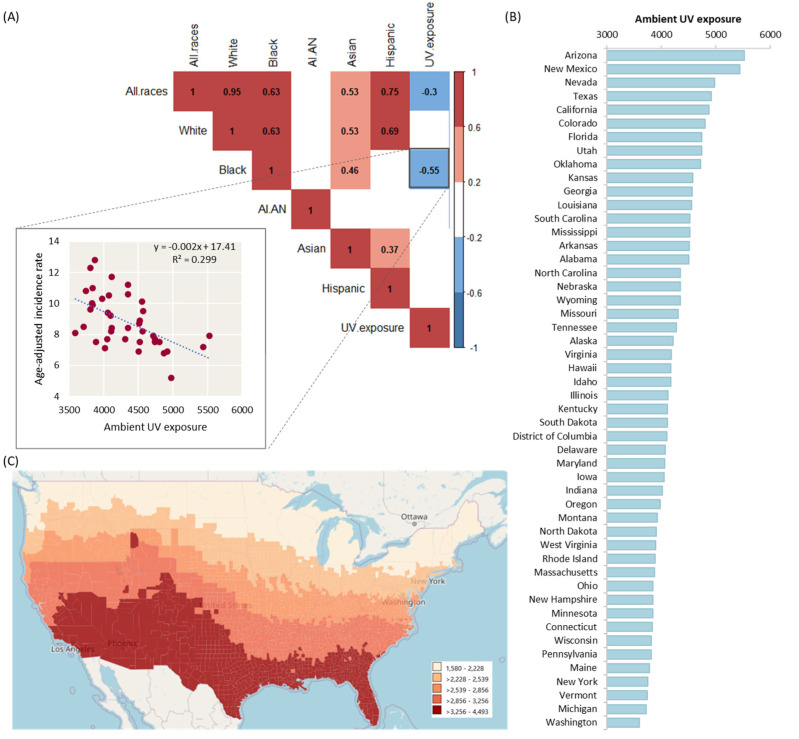
**Ultraviolet exposure varies by state.** (**A**) Correlation matrix for UV exposure with age-adjusted incidence rates of thyroid cancer of all races and each race individually. Pearson’s correlation was used. Correlation coefficients of significant results are shown only. UV exposure was negatively correlated with incidence rate of thyroid cancer for overall population (r = −0.299, *p* = 0.035) and Black population (r = −0.555, *p* < 0.001). (**B**) Average UV exposure (Watt-Hours Per Square Meter) within the United States. (**C**) Map for ultraviolet exposure across the US counties. Data source: The CDC National Environmental Public Health Tracking Network (1011–2015) (https://ephtracking.cdc.gov/DataExplorer/?c=26: accessed on 1 January 2021).

UV exposure was significantly negatively correlated with thyroid cancer incidence rates when analyzed across all races (r = −0.299, *p* = 0.035) (Figure 2A–C). When stratified by patient race, UV exposure was negatively correlated with thyroid cancer rates in Black populations specifically (r = −0.55, *p* < 0.001). All nine states (Massachusetts, Pennsylvania, Kentucky, Nebraska, Ohio, New York, Wyoming, Delaware, Oregon) with the highest incidence rate of all-stage thyroid cancer in Black populations had relatively low total UV exposure rates. In contrast, seven of the eight states (Nevada, California, Alabama, Texas, New Mexico, Colorado, and Mississippi) with the lowest incidence rate of all-stage thyroid cancer in Black populations had relatively high total UV exposure rates.

**Figure 3:**
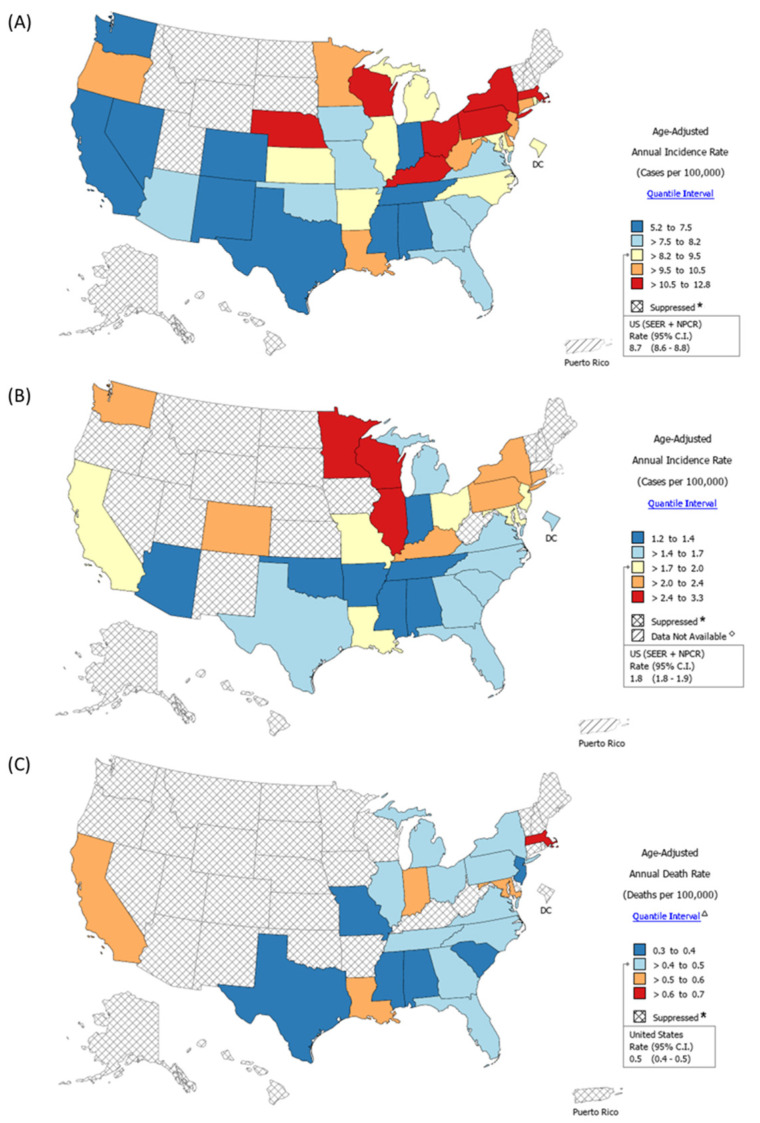
**Geographic maps for the incidence and mortality rates of thyroid cancer in Black populations.** (**A**) Age-adjusted incidence rate in all stages (2014–2018) in Black populations. (**B**) Age-adjusted incidence rate in late stages (2014–2018) in Black populations. (**C**) Mortality rate (2015–2019) in Black populations. Data for counts fewer than 16 records in a specific area–sex–race category have been suppressed to ensure confidentiality and stability of rate estimates. * Data for counts fewer than 16 records in a specific area-sex-race category has been suppressed to ensure confidentiality and stability of rate estimates per guidelines.

**Figure 4:**
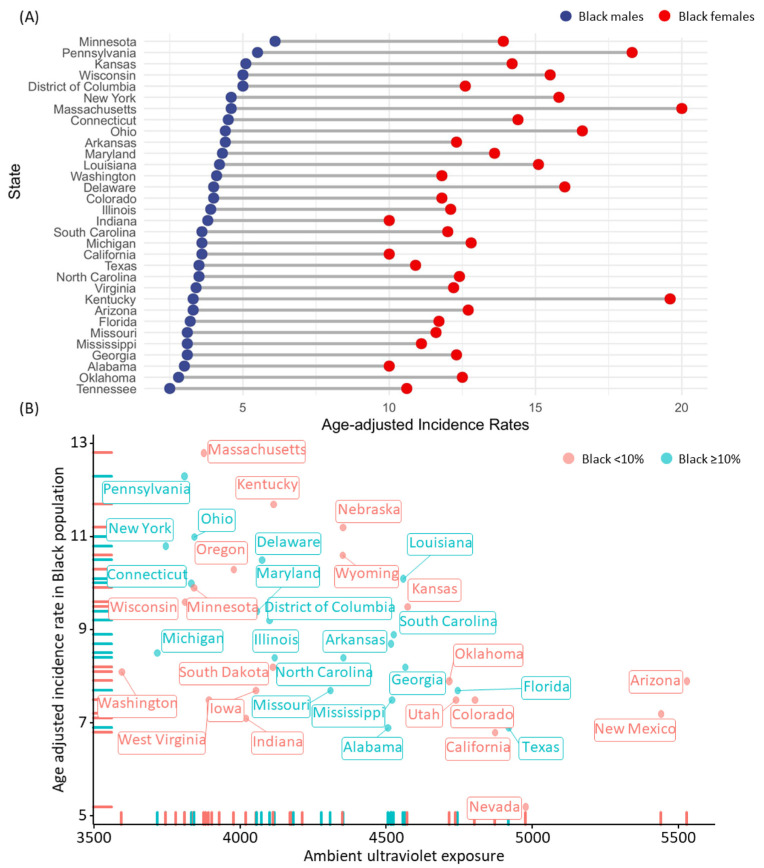
**Thyroid cancer incident rates across states and UV exposure in Black populations.** (**A**) Age-adjusted incidence rates of thyroid cancer in Black males and Black females. Data for counts fewer than 16 records in a specific area–sex–race category have been suppressed to ensure confidentiality and stability of rate estimates. States without paired data for the other sex were removed. (**B**) Association between the amount of UV exposure and age-adjusted incidence ratio stratified by the percentage of Black people across the United States.

This trend finding is depicted in Figure 3, where thyroid cancer all-stage incidence (Figure 3A), thyroid cancer late-stage incidence (Figure 3B), and thyroid cancer mortality rate (Figure 3C) all suggest increased rates in northern states (in general, decreased UV exposure) and decreased rates in southern states (in general, increased UV exposure). This trend persisted when thyroid cancer incidence rate per state in Black patients were stratified by sex (Figure 4A). Interestingly, a scatterplot analysis to determine if the percent of the state population which was Black influenced this trend (stratified at <10% or ≥10%) could not elicit a trend (Figure 4B). Additionally, subsequent multivariate analysis determined UV exposure to be the only protective factor against developing thyroid cancer (beta coefficient = −0.002, 95% CI: −0.003 to −0.001, *p* = 0.006). The incidence rates of thyroid cancer in Black populations stratified by sex and each state can be found in Supplementary Table S1.

## 5. Discussion

Thyroid cancer is the most common endocrine malignancy [1,2]. While risk factors, as well as protective factors, of thyroid cancer development have been studied extensively [20,21], the influence of UV exposure is still poorly understood. To our best knowledge, this is the first study to examine the influence of UV exposure on thyroid cancer incidence stratified by race.

Our work found that thyroid cancer incidence rates have declined in recent years. Though increased imaging studies allowing for increased diagnostic scrutiny have allowed detection rates to consistently rise for the past four decades [22], the rate has begun to plateau and now fall. Thyroid cancer incidence rates between the years of 2014 and 2016 in the United States remained stable [19]. Expanding on this, Lee et al. found that detection rates fell between the years of 2015 and 2017 [23] and Megwalu et al. found this consistent with data reported from the years of 2015 to 2018 [18]. Interestingly, the latter work noted a decreased incidence in tumors ≤1.0 cm (which are the most common thyroid tumors) [18], while our work found a greater decline in detection rates for females (as opposed to males). Together, this may suggest a decrease in the detection of small, clinically insignificant incidentalomas in women which would be identified during screening procedures.

Thyroid cancer presents heterogeneously with respect to race. A 2015 epidemiology study found that thyroid cancer presented most commonly in White patients [24]. This finding could be explained by the increased incidence of autoimmune conditions in White populations, such as Hashimoto’s thyroiditis [20,25,26], and the direct correlation of benign thyroid disease (such as Hashimoto’s thyroiditis) and thyroid cancer [20,25]. We found that the incidence rates of thyroid cancer were highest in Asian/Pacific Islanders and White populations and lowest in Black and Native American populations, with Black populations having had the steepest decline in recent years. Despite having the lowest incidence rate, Black men had the worst 5-year survival rates in distant metastatic thyroid cancer cases. Possible causes for this decrease in cancer rate but increase in poor prognosis affecting Black men include socioeconomic barriers that prevent access to quality healthcare services, including doctors’ appointments, proper imaging, medication, radiotherapy, and treatment procedures, among others [27,28]. This hypothesis is supported by the work of Ghori et al., who found that ten-year thyroid cancer survival rates were lower in those of the lowest income quartile as well as males [29]. Additionally, their finding that males were more likely than females to present at later stages of cancer suggests a possible explanation for why Black males had lower survival rates than Black females in our work. As comorbidities were recently demonstrated to occur in as many as two-thirds of cancer patients, another possible cause includes sex-based differences in rates of comorbidities [30]. Though his area warrants further investigation, controlling for factors such as socioeconomic status and sex-based differences, the success of assisting underserved Black male communities has been rather successful [31,32,33] and may be a potential first-step in for addressing the disparity in thyroid cancer survival rates among Black men.

Our findings also found a strong correlation between UV exposure and decreased thyroid cancer incidence, suggesting UV light to serve as a protective measure against the development of thyroid cancer (r = −0.299, *p* = 0.035). Moreover, this protection was found to be heightened in Black populations. This aligns with the study by Zamoiski et al., which demonstrated that increased UV light exposure was negatively correlated with thyroid cancer rates in patients with benign thyroid conditions and those with darker complexions [5]. Our findings suggest that UV exposure was significantly protective against thyroid cancer in all racial groups (and therefore all complexions). Additionally, this association was most pronounced in Black populations, suggesting a link between skin pigmentation and the protective effects of UV exposure. Corroborating our work, studies from Spain [34] and China [35] both report a negative correlation between UV exposure and thyroid cancer risk. This could be potentially be explained by one study which reported vitamin D supplementation to be serve as a protective factor against thyroid cancer [36]. As aforementioned, exposure to UV sunlight increases the conversion of precursor vitamin D into its active counterpart, calcitriol, which has an inhibitory effect on carcinogenesis [37,38], tumor progression [39], cell growth [38,40], and angiogenesis [40]. The lack of UV exposure results in diminished levels of vitamin D, which has been implicated in the pathophysiology of several solid cancers, including those of the female breast [10,11], prostate [8,9], colorectal [41,42], pancreatic [43,44], lung [45], ovarian [12], and stomach [46,47]. Still, these findings are not generalizable to all cancers [13,48] and warrants further investigation. While our work found thyroid cancer incidence to be negatively correlated with total UV light exposure, it is important to consider patient-specific details before generalizing conclusions to states and large populations. For example, within a single state, an individual whose occupation exposes them sunlight, such as a brick layer or a construction worker, may have a significantly different overall exposure time to UV light than an office worker. Future works should elucidate these patient-specific details so as to better understand the applicability and generalizability of the inverse correlation between UV exposure and thyroid cancer incidence, specifically based on patient occupation and/or outdoor activity. For now, the potentially protective effect of UV exposure cannot be advertised as beneficial to all patients.

Finally, we acknowledge that our study is not without limitation. Though the SEER database allows the analysis of an impressive sample size, the database is retrospective in nature and lacks pertinent parameters, such as patient comorbidities or common sex-based differences which could allow further insight. Yet, its national representation allows the data to be generalizable to thyroid cancer patients of any race. In addition, the correlational nature of this study, like all epidemiological works concerning this topic, do not reflect a direct causal effect of UV exposure. Still, our work analyzed over 284,000 patients across the United States and suggests a protective effect of UV exposure. Future studies incorporating patient-specific details, such as patient occupation and outdoor exposure, are needed to elucidate and further generalize our conclusions.

## 6. Conclusions

Overall, the incidence of thyroid cancers has recently decreased in the United States, and most steeply in the Black population. Interestingly, UV exposure may be a protective factor against the incidence of thyroid cancer.

## Figures and Tables

**Table 1:** Incident rate and trends of all stage thyroid carcinoma by race (2014–2018).

Characteristics		Age-Adjusted Incidence Rate Cases per 100,000	Average Annual Count	Recent 5-Year Trend in Incidence Rates
**All races**	**Overall**	14.1 (14.0, 14.1)	47,728	−2.9 (−4.1, −1.7) *
	**Male**	7.3 (7.2, 7.3)	12,303	−1.9 (−3.3, −0.5) *
	**Female**	20.7 (20.6, 20.8)	35,425	−3.1 (−4.4, −1.8) *
**White**	**Overall**	14.7 (14.6, 14.7)	39,484	−3.1 (−4.3, −1.8) *
	**Male**	7.7 (7.6, 7.7)	10,580	−1.9 (−3.4, −0.4) *
	**Female**	21.7 (21.5, 21.8)	28,904	−3.4 (−4.7, −2.2) *
**Black**	**Overall**	8.7 (8.6, 8.8)	3745	−5.4 (−7.7, −3.2) *
	**Male**	3.8 (3.6, 3.9)	727	−4.3 (−6.7, −1.9) *
	**Female**	13.0 (12.8, 13.2)	3018	−5.1 (−7.6, −2.6) *
**American Indian/Alaskan Native**	**Overall**	8.0 (7.6, 8.4)	332	−0.7 (−3.6, 2.4)
	**Male**	3.8 (3.4, 4.2)	73	3.0 (1.0, 5.1) #
	**Female**	12.2 (11.5, 12.9)	259	−0.3 (−3.9, 3.4)
**Asian or Pacific Islander**	**Overall**	14.9 (14.7, 15.2)	3156	−1.5 (−2.5, −0.4) *
	**Male**	7.3 (7.0, 7.5)	700	−1.8 (−3.7, 0.2)
	**Female**	21.7 (21.3, 22.1)	2456	−1.8 (−3.1, −0.4) *
**Hispanic**	**Overall**	13.4 (13.2, 13.5)	6536	−0.9 (−2.7, 1.0)
	**Male**	5.8 (5.6, 5.9)	1286	1.4 (0.3, 2.5) #
	**Female**	20.9 (20.7, 21.2)	5250	−1.0 (−3.0, 1.0)

Data are reported as rate (95% confidence interval). Table demonstrates race-and sex-stratified differences in thyroid cancer counts, incidence rates, and trends. In the 5-year trend in the incident rates column, (*) indicates a significant decrease, whereas (#) indicates a significant increase.

## Data Availability

Data are contained within the article or supplementary material.

## Supplementary Materials

The supplementary material can be downloaded at https://www.mdpi.com/article/10.3390/biomedicines10102452/s1. Supplementary Table S1. Incidence rates of thyroid cancer in Black populations stratified by sex and each state.
